# Isolation and genotypic characterization of extended-spectrum beta-lactamase-producing *Escherichia coli* O157:H7 and *Aeromonas hydrophila* from selected freshwater sources in Southwest Nigeria

**DOI:** 10.1038/s41598-023-38014-y

**Published:** 2023-07-03

**Authors:** Mary A. Bisi-Johnson, Atilade A. Adedeji, Adebayo A. Sulaiman, Martins A. Adefisoye, Anthony I. Okoh

**Affiliations:** 1 Department of Microbiology, Obafemi Awolowo University, Ile-Ife, Nigeria; 2 Department of Microbiology, School of Science and Technology, Babcock University, Ilishan-Remo, Nigeria; 3 Applied and Environmental Microbiology Research Group, Department of Biochemistry and Microbiology, University of Fort Hare, Alice, 5700 South Africa; 4 Department of Environmental Health Sciences College of Health Sciences, University of Sharjah, Sharjah, United Arab Emirates

**Keywords:** Microbiology, Molecular biology, Environmental sciences

## Abstract

The proliferation of antibiotic-resistant bacteria and antimicrobial resistance is a pressing public health challenge because of their possible transfer to humans via contact with polluted water sources. In this study, three freshwater resources were assessed for important physicochemical characteristics as well as heterotrophic and coliform bacteria and as potential reservoirs for extended-spectrum beta-lactamase (ESBL) strains. The physicochemical characteristics ranged from 7.0 to 8.3; 25 to 30 °C, 0.4 to 93 mg/L, 0.53 to 8.80 mg/L and 53 to 240 mg/L for pH, temperature, dissolved oxygen (DO), biological oxygen demand (BOD_5_) and total dissolved solids, respectively. The physicochemical characteristics mostly align with guidelines except for the DO and BOD_5_ in some instances. Seventy-six (76) *Aeromonas hydrophila* and 65 *Escherichia coli* O157: H7 isolates were identified by preliminary biochemical analysis and PCR from the three sites. Among these, *A. hydrophila* displayed higher frequencies of antimicrobial resistance, with all 76 (100%) isolates completely resistant to cefuroxime and cefotaxime and with MARI ≥ 0.61. The test isolates showed more than 80% resistance against five of the ten test antimicrobials, with resistance against cefixime, a cephalosporin antibiotic being the highest at 95% (134/141). The frequency of the detection of the resistance genes in the *A. hydrophila* isolates generally ranged between 0% (*bla*_SHV_) and 26.3% (*bla*_CTX-M_), while the frequency of detection among the *E. coli* O157:H7 isolates ranged between 4.6% (*bla*_CTX-M_) and 58.4% (*bla*_TEM_). Our findings indicate that the distribution of antibiotic-resistant bacteria with diverse ESBL-producing capabilities and virulence genes in freshwater sources potentially threatens public health and the environment.

## Introduction

Easy access to clean and safe water supply as well as adequate sanitation, are fundamental human rights and one of the sustainable development goals (SDGs) of the United Nations General Assembly to be achievable by 2030. Inadequate sanitation and polluted water sources affront human dignity and can lead to disease outbreaks, lower productivity, exorbitant medical costs, increased waterborne illnesses and death rates^1,2^. Surface waters are “lifelines,” providing water for the immediate needs of communities settled along their courses. Freshwater sources, however, are frequently subjected to pollution with microbial and biochemical pollutants from point and nonpoint sources. Remarkably, indicator microbes, such as faecal coliforms and *E. coli*, have been used as surrogates for the probable presence of pathogenic organisms in pollution monitoring and control^3^. Some clinically significant pathogens, as well as established aetiological agents of diarrhoea and other gastrointestinal illnesses, are frequently isolated from rural dwellers of most developing countries due to contact with polluted water sources and inadequate sanitation^4^.

The emergence and re-emergence of water-related infectious diseases exert massive pressure on the water-food-public health interface, resulting in rising incidences of morbidity and mortality in many developing and underdeveloped nations. Understanding the transmission routes of the disease agents has become a priority area for research institutions at local, national, and international levels^5^.

*Escherichia coli* O157:H7 and *Aeromonas hydrophila* are emerging pathogens increasingly recognised as significant aetiological agents of acute enteric infection, including diarrhoea^6^. *E. coli* O157:H7 is an aetiological agent of severe and bloody diarrhoea and other serious complications, including haemolytic-uremic syndrome (HUS), making this bacterium a pathogen of public health priority^7,8^. Ground beef and other meat products have been implicated as the predominant transmission route of *E. coli* O157:H7 to humans. However, sporadic outbreaks have been linked to contaminated water sources, including drinking water, irrigation water, and wastewater discharges^9,10^. Antimicrobial resistance development in pathogenic strains of *E. coli*, including *E. coli* O157:H7, has been linked to the misuse of antibiotics. The resistant strains harbour resistance genes, such as *blaSHV*, *cat1* and *sul1*, among others, conferring resistance to commonly used antimicrobials. Likewise, aeromonads have been commonly isolated from food products and aquatic environments^11,12^. The continuous emergence of *A. hydrophila* as an important human pathogen, its ability to acquire virulence factors and become resistant to multiple antibiotics, and its involvement in extra-intestinal and systemic infections make *A. hydrophila* an important pathogen of public health concern^13^.

The aquatic milieu presents a favourable niche for the evolution and spread of multidrug-resistant pathogens with extraordinary virulence capabilities^14^. Wastewater treatment plants, in particular, have been identified as significant bioreactors where antibiotic-resistant bacteria (ARB) and their associated resistance determinants are enriched and subsequently discharged into surface water environments with their attendant public health hazards^15–17^.

Extended-spectrum beta-lactamases (ESBL)-producing *A. hydrophila* strains have been reported mainly in Europe, especially in France^18^, Poland^19^, Portugal^20^ and other regions^21,22^. Similarly, rising incidences of resistant *E. coli* strains against established antimicrobials, such as extended-spectrum beta-lactams, complicate therapeutic interventions^23^. There is a dearth of documented information on the genotypic profiles of aetiological agents of acute gastroenteritis, such as *Aeromonas* sp. and *E. coli* O157:H7, from the aquatic milieu of the southwestern part of Nigeria.

The current study aimed to assess selected physicochemical and bacteriological qualities of three important freshwater resources in the southwestern region of Nigeria. Furthermore, to characterise ESBL-resistant *E. coli* O157:H7 and *Aeromonas* sp. and their virulence-modulating genes from three watershed using phenotypic and genotypic techniques.

## Materials and methods

### Study area description

**Figure 1:**
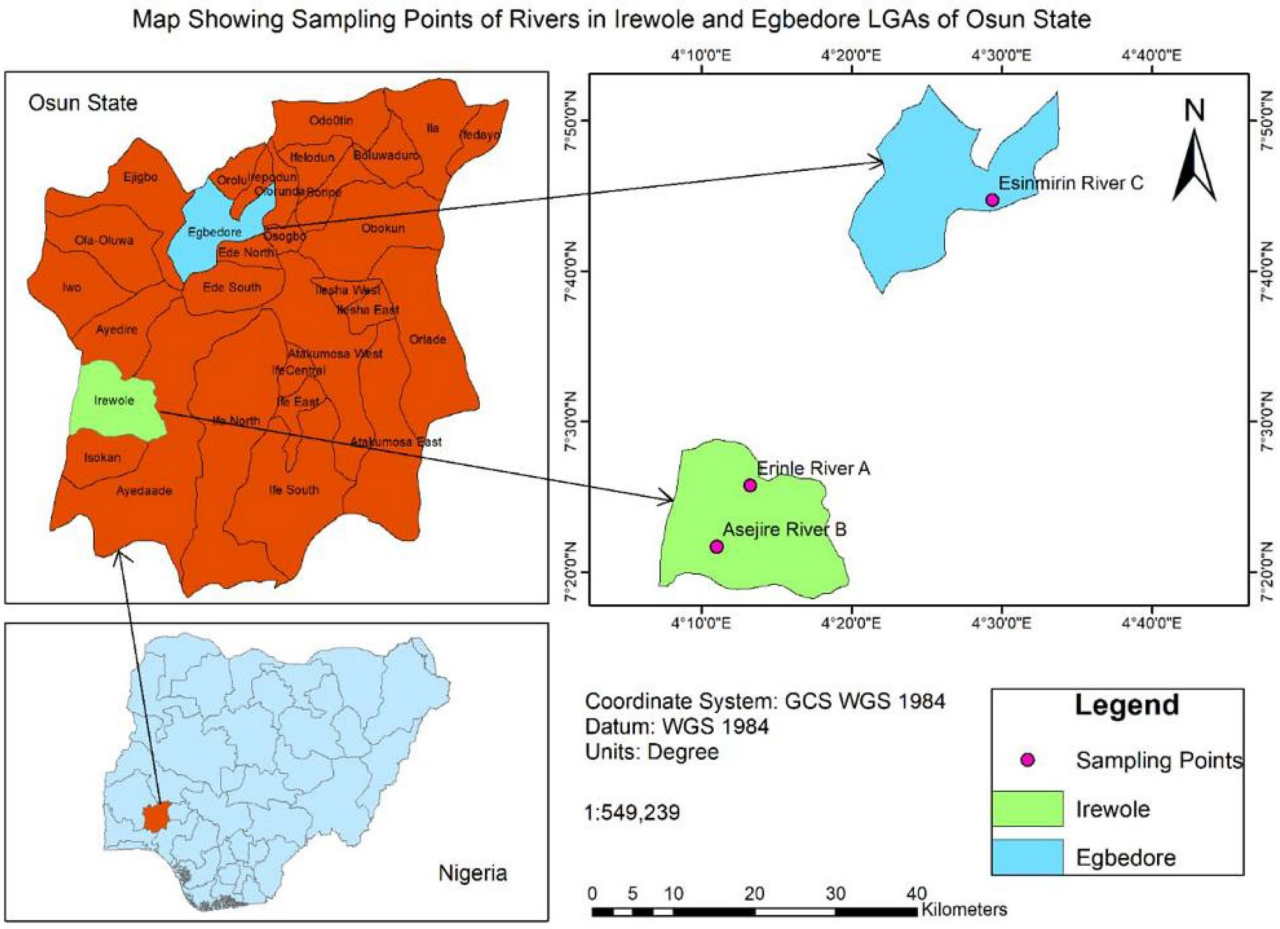
The map showing the locations of the study sites in Southwest Nigeria. The map was created using: ArcGIS [GIS software]. Version 10.0. Redlands, CA: Environmental Systems Research Institute, Inc., 2010. (https://www.esri.com/en-us/home).

Initial reconnaissance survey and mapping of the study area were done after the State Ministries of Environment, Water Resources, Lands, and Housing had been duly consulted. The study sites included three rivers (Erinle River, Asejire River and Esimirin Rivers) which fall within three local governments areas of Osun State in the South-western geopolitical zone of Nigeria with GPS coordinates 7°25′44″ N, 4°13′14″ E; 7°21′40″ N, 4°11′00″ E and 7°44′44″ N; 4°29′22″ E respectively (Fig. 1). Factors considered in the selection of the study sites include anthropogenic activities, domestic, agricultural, and industrial activities along the watercourse, sanitary states and the economic importance of the rivers. For ease of reference, the study sites are coded as River-A: Erinle River, River-B: Asejire River and River-C: Esimirin River, while the descriptions of the study sites are depicted in Table 1.

**Table 1:** Detail description of sampling sites.

Study sites (codes)	GPS coordinates	Anthropogenic activities around study locations
Erinle (River-A)	7° 25′ 44″ N, 4° 13′ 14″ E	Tourism, crop irrigation, livestock farming, and fishing. Settlements along the course of the river source water for domestic uses, such as laundry and cooking. Also, serve as abstraction water for a portable water treatment plant
Asejire (River-B)	7° 21′ 40″ N, 4° 11′ 00″ E	Fishing, irrigation, livestock farming and domestic activities, including laundry, cooking and water abstraction. They are also used as waste drainage by a soft drink bottling industry
Esinmirin (River-C)	7° 44′ 44″ N, 4° 29′ 22″ E	Fishing, crop farming, animal husbandry, domestic water sources, small-scale industries (such as brick and concrete) source water from the river and release their waste into it. Traditional and religious activities

### Water sampling for physicochemical parameters

Water samples for the physicochemical characteristics of the rivers were collected fortnightly from each of the study sites over a six-month sampling period. Some critical physicochemical characteristics were determined in situ. These include the use of Crison Multimeter MM40 (Crison Instrument S.A., Barcelona, Spain) for water pH, temperature (temp °C), total dissolved solids (TDS mg/L), and turbidity by microprocessor turbidity meter (HACH Company, model 2100P). Dissolved oxygen (DO) was determined by HQ40d BOD meter (HACH Company, Loveland, C, USA), while the biological oxygen demand (BOD_5_) was determined in the laboratory after five days of incubation in a dark cupboard^24^. All analyses were done in triplicates, and values were reported as means ± standard error of means.

### Sample collection and microbiological analysis

One hundred and eight (108) river water samples were aseptically collected in sterile 1 L glass bottles and transported in cooler boxes (4 °C), and processed upon arrival at the Microbiology laboratory (Department of Microbiology, Obafemi Awolowo University, Nigeria). Samples were collected fortnightly over six months, divided into the raining (July to September) and the dry (October to December) seasons. The collected samples were subjected to bacteriological analysis to determine heterotrophic plate count (HPC), total coliforms (TC), faecal coliforms (FC), *A. hydrophila*, and *E. coli* O157:H7 counts. Enumeration of the presumptive bacteria was done by making appropriate serial dilution of the samples and spread plating 100 µL aliquots of the diluted samples on appropriate media in triplicate. All media used for the enumerations were supplied by Laboratorios CONDA (Madrid, Spain). For the enumeration of HPC densities, the diluted samples were spread on R2A Agar (Laboratorios CONDA, Madrid, Spain) and incubated at 37 °C for 24 h, while m-FC Agar (incubated at 44.5 °C for 24 h) and Endo Agar Base (incubated at 37 °C for 24 h) were used for the enumeration of FC and TC respectively. *Aeromonas* Agar Base (Ryan) (Laboratorios CONDA, Madrid, Spain) supplemented with ampicillin and incubated at 37 °C for 24 h was used enumerate and isolate *A. hydrophila* while *E. coli* O157:H7 was enumerated using *E. coli* O157:H7 Chromogenic Agar Base (Laboratorios CONDA, Madrid, Spain) incubated at 37 °C for 24 h. The bacterial counts were done in triplicate and reported as cfu/100 mL. Distinct presumptive colonies of *A. hydrophila* (green with a black centre) and *E. coli* O157:H7 (pale pink) were picked from the enumeration plates and purified further in the respective medium until pure cultures were obtained. The pure cultures were stored on nutrient agar (NA) slants and refrigerated at 4 °C for further analyses.

### Isolates biochemical identification

Freshly grown cultures of presumptive isolates were identified by a panel of biochemical tests including Gram staining, spore staining, catalase, starch hydrolysis, gelatin liquefaction, nitrate reduction, methyl red, vogues-proskauer, motility-indole-ornithine, H_2_S, sugar fermentation, triple sugar iron agar, citrate utilization, oxidase, and urease tests.

### Antibiotic susceptibility profiling of isolates

Antibiotic susceptibility patterns of *A. hydrophila* and *E. coli* O157:H7 were assayed using the disc diffusion method on freshly prepared Muller Hinton Agar plates, and the results were interpreted according to the Clinical and Laboratory Standard Institute^25^. The susceptibility profiles of the isolates were established against a panel of nine commercially available antibiotics. The antibiotics used in the study include amoxicillin (25 µg); augmentin (10 µg); cefixime (5 µg); cefotaxime (30 µg); ceftazidime (30 µg); cefuroxime (30 µg); ciprofloxacin (5 µg); gentamicin (10 µg); nitrofurantoin (30 µg) and ofloxacin (5 µg).

### Determination of isolates multiple antibiotic resistance indices (MARI)

The MARIs of isolates were estimated based on their phenotypic antibiotic resistance patterns^26^. Multiple antibiotic resistance (MAR) was defined in the study as resistance to three or more test antibiotics. The formula used for the MARI estimation is as follows:

$$ {\text{MARI}} = \frac{{{\text{No}}.\;{\text{of}}\;{\text{antibiotics}}\;{\text{to}}\;{\text{which}}\;{\text{isolates}}\;{\text{are}}\;{\text{resistant}}}}{{{\text{No}}.\;{\text{of}}\;{\text{antibiotics}}\;{\text{tested}}\; \times \;{\text{No}}.\;{\text{of}}\;{\text{isolates}}}} $$

### Phenotypic detection of ESBL production in A. hydrophila and E. coli O157:H7 isolates

The double-disc synergy test was employed to characterise the phenotypic production of ESBL, according to CLSI (2018). Briefly, freshly grown cultures were standardized (0.5 McFarland) and spread evenly to form a lawn culture on Muller Hinton agar plates. An antibiotic disc containing amoxicillin/clavulanic (20/10 µg) acid was carefully placed at the centre of the plates while two discs of third-generation cephalosporin (e.g., ceftriaxone, cefotaxime or ceftazidime) antibiotics were carefully placed at either 15 mm or 20 mm apart respectively, centre-to-centre to that of the amoxicillin/clavulanic acid disc. The plates were incubated inverted at 37 °C for 24 h. The positive result for ESBL production was taken as any distortion or increase in the zone toward the amoxicillin/clavulanic disc.

### Molecular characterisation of ESBL producing genes of isolates

Total genomic DNA was extracted from freshly grown cultures by boiling method^27^. The DNA extracts were stored on ice and used for PCR immediately to detect ESBL-producing genes, including *bla*_CTX-M_, *bla*_SHV*,*_ and *bla*_TEM_. Amplifications were carried out in a final reaction volume of 25 µL consisting of 12.5 µL 2X PCR Master Mix Thermo Scientific Inc., Waltham, MA, USA), 1.0 µL each of forward and reverse primers (10 pmol), 5.5 µL of nuclease-free water and 5.0 µL of DNA template. The specific primers and the annealing temperatures are shown in Table 2. The amplicons were resolved on 1.5% agarose gel electrophoresis stained with 0.5 mg/L ethidium bromide with 0.5 × TBE buffer and visualized under the wave ultraviolet transilluminator and photographed using gene gel bioimaging system.

**Table 2:** Primers used in this study for specific genes amplification.

Primers	Sequences 5′–3′	Target genes	Product sizes (bp)	Annealing temperatures	References
CTX-M F CTX-M R	CGATGTGCAGTACCAGTAA TTAGTGACCAGAATAAGCGG	*bla*_CTX-M_	585	60	^30^
TEM F TEM R	CCCCGAAGAACGTTTTC ATCAGCAATAAACCAGC	*bla*_TEM_	517	51	^30^
SHV F SHV R	AGGATTGACTGCCTTTTTG ATTTGCTGATTTCGCTCG	*bla*_SHV_	393	56	^30^
Lip F Lip R	ATCTTCTCCGACTGGTTCGG CCGTGCCAGGACTGGGTCTT	Lipase	383–389	52	^31^
Ela F Ela R	ACACGGTCAAGGAGATCAAC CGCTGGTGTTGGCCAGCAGG	Elastase	540	58	^31^
hylA F hlyA R	CAATAGTGCCAAAGCCGAAT TCCAGCACCACAACGAGAAT	Haemolysin	496	60	^32^
rfbE _O157_F rfbE _O157_R	CTACAGGTGAAGGTGGAATGG ATTCCTCTCTTTCCTCTGCGG	rfbE_O157_	327	58	^33^
flic_H7_F flic_H7_R	GCGCTGTCGAGTTCTATCGAGC CAACGGTGACTTTATCGCCATTCC	flic_H7_	625	52	^33^

### Molecular detection of virulence genes in A. hydrophila and E. coli O157:H7 isolates

Virulence genes were detected in some of the bacterial isolates by PCR using the primers shown in Table 2. The lipase and elastase genes were targeted in *A. hydrophila*^28^, while the *fliC*_H7_, *rfbE*_O157_ and *hly* gene (α-haemolysin production) were targeted in *E. coli* O157:H7 strains^29^. For quality control and to ensure the specific amplification of the target genes, molecular grade reagents purchased from Inqaba Biotec Pty Inc., South Africa, were used for all molecular analysis. DNA extract from the ATCC 7966 type cultures was used as positive controls for *Aeromonas* species amplification, while a previously PCR-confirmed strain of *E. coli* O157:H7 from the culture collection of the Applied and Environmental Microbiology Research Group (AEMREG), University of Fort Hare, South Africa was used as the positive control for *E. coli* O157:H7 amplifications. The negative controls included mixtures of all the molecular reagents without a DNA template.

### Statistical analysis

The student t-test was used to compare the means of physicochemical and microbiological parameters for the two seasons. One-way analysis of variance and Pearson correlation test was used to compare the relationship between physicochemical and microbiological parameters for the three sites. The confidence limit was set at *p* ≤ 0.05 for all analyses.

## Results

### Physicochemical parameters

The physicochemical characteristics of the three rivers assessed in this study are presented in Table 3, and the seasonal comparison of the characteristic is in Table 4. The data shows the mean values of triplicate determinations ± the standard error. The parameters evaluated were generally compared to the stipulated standards for drinking water by the Standard Organisation of Nigeria^34^ and the World Health Organisation^35^ international standards for drinking water (Table 3).

**Table 3:** The mean values of the physicochemical parameters of samples analysed at the three study sites.

	Parameters	July ’18		August ’18		September ’18		October ’18		November ’18		December ’18		FMEnv, 2002	SON, 2007	WHO, 2011
		1st	2nd	1st	2nd	1st	2nd	1st	2nd	1st	2nd	1st	2nd			
River-A	pH	7.0 ± 0	7.1 ± 0	7.2 ± 0.1	7.2 ± 0	7.3 ± 0.1	7.2 ± 0	7.2 ± 0.1	7.3 ± 0	7.5 ± 0	7.6 ± 0.1	7.7 ± 0	7.8 ± 0	6.5–8.5	6.5–8.5	6.5–9.5
	Temp (°C)	25 ± 0	27 ± 1	28 ± 1	25 ± 0	26 ± 1	29 ± 1	30 ± 1	30 ± 0	31 ± 0	32 ± 1	32 ± 1	32 ± 0	< 30 °C	Ambient	25–30
	TDS (mg/L)	82 ± 2	85 ± 5	98 ± 2	118 ± 5	144 ± 0	118 ± 2	121 ± 2	132 ± 2	136 ± 4	139 ± 3	143 ± 6	145 ± 4	< 500	100	1000
	DO (mg/L)	3.2 ± 0.5	4.3 ± 0.6	5.7 ± 0.1	5.1 ± 0.2	6.5 ± 0.4	6.1 ± 0.4	0.9 ± 0.1	1.4 ± 0.2	0.9 ± 0.3	1.1 ± 0.5	0.7 ± 0.1	0.4 ± 0	5	NA	≥ 5
	BOD (mg/L)	0.53 ± 0.13	1.00 ± 0.20	2.80 ± 0.00	1.40 ± 0.10	1.73 ± 0.35	1.60 ± 0.23	2.00 ± 0.23	3.05 ± 0.13	3.68 ± 0.16	5.58 ± 0.92	6.65 ± 0.55	7.73 ± 0.53	NA	6	6
River-B	pH	7.5 ± 0.1	7.1 ± 0.1	7.1 ± 0.1	7.0 ± 0.1	7.0 ± 0	7.0 ± 0.1	7.1 ± 0.1	7.2 ± 0.1	7.8 ± 0.1	7.8 ± 0.1	8.1 ± 0	8.3 ± 0	6.5–8.5	6.5–8.5	6.5–9.5
	Temp (°C)	25 ± 0	29 ± 0	27 ± 0	28 ± 0	26 ± 0	26 ± 0	29 ± 0	30 ± 0	29 ± 0	30 ± 0	30 ± 0	32 ± 1	< 30 °C	Ambient	25–30
	TDS (mg/L)	79 ± 1	67 ± 2	65 ± 1	61 ± 1	53 ± 0	53 ± 0	67 ± 2	69 ± 1	69 ± 1	81 ± 1	110 ± 3	112 ± 2	< 500	100	1000
	DO (mg/L)	2.1 ± 0.1	3.3 ± 0.6	9.3 ± 0.5	7.2 ± 1.4	8.3 ± 0.1	1.3 ± 0.1	2.4 ± 0.3	2.4 ± 0.1	2.3 ± 0.1	2.2 ± 0.3	1.7 ± 0.2	1.3 ± 0.1	5	NA	≥ 5
	BOD (mg/L)	0.66 ± 0.26	0.53 ± 0.13	2.08 ± 1.22	3.46 ± 1.27	4.53 ± 0.26	0.64 ± 0.12	1.46 ± 0.13	3.73 ± 0.13	4.60 ± 0.41	6.66 ± 0.63	8.40 ± 0.69	8.80 ± 0.40	NA	6	6
River-C	pH	7.4 ± 0.1	7.2 ± 0	7.1 ± 0	7.3 ± 0.1	7.2 ± 0	7.3 ± 0	7.6 ± 0.1	7.5 ± 0.1	7.6 ± 0	7.9 ± 0	7.7 ± 0	7.9 ± 0	6.5–8.5	6.5–8.5	6.5–9.5
	Temp (°C)	27 ± 0	26 ± 0	26 ± 0	24 ± 1	25 ± 1	26 ± 0	28 ± 0	28 ± 1	28 ± 0	28 ± 1	29 ± 0	30 ± 0	< 30 °C	Ambient	25–30
	TDS (mg/L)	124 ± 2	121 ± 1	120 ± 2	239 ± 2	212 ± 2	200 ± 2	210 ± 3	212 ± 2	202 ± 1	207 ± 5	224 ± 4	240 ± 4	< 500	100	1000
	DO (mg/L)	2.5 ± 0.1	3.3 ± 0.1	2.1 ± 0.1	6.2 ± 1.2	3.1 ± 0.1	3.7 ± 0.3	1.9 ± 0.1	1.6 ± 0	1.5 ± 0.1	1.3 ± 0.4	1.1 ± 0.1	0.9 ± 0.1	5	NA	≥ 5
	BOD (mg/L)	0.93 ± 0.26	0.93 ± 0.13	0.53 ± 0.13	1.76 ± 0.99	1.20 ± 0.23	1.33 ± 0.35	2.53 ± 0.13	2.66 ± 0.13	2.80 ± 0.11	3.00 ± 0.11	3.29 ± 0.13	3.58 ± 0.23	NA	6	6

Values represent the mean ± standard error of mean (n = 3).

*TDS* total dissolved solids, *DO* dissolved oxygen, *BOD*_*5*_ biological oxygen demand after a 5-day incubation period, *FMEnv* Federal Ministry of Environment, *SON* Standard Organisation of Nigeria, *WHO* World Health Organisation.

**Table 4:** Comparison of the physicochemical characteristics for wet and dry seasons at the three study sites.

SEASON	Study site	Physicochemical characteristic				
		pH	Temperature (°C)	TDS (mg/L)	DO (mg/L)	BOD (mg/L)
WET	River-A	7.2 ± 0.0	26.7 ± 0.7	107.5 ± 2.67	5.15 ± 0.37	1.51 ± 0.17
	River-B	7.12 ± 0.0	26.8 ± 0.0	63.0 ± 0.83	5.25 ± 0.47	1.98 ± 0.54
	River-C	7.25 ± 0.0	25.7 ± 0.33	169.3 ± 1.83	3.48 ± 0.32	1.11 ± 0.35
	F	1.408	2.303	14.44	1.176	0.939
	P	0.275	0.134	0.0003	0.3355	0.413
DRY	River-A	7.52 ± 0.03	31.2 ± 0.5	136 ± 3.5	0.9 ± 0.2	4.78 ± 0.42
	River-B	7.72 ± 0.07	30.0 ± 0.17	84.7 ± 1.67	2.05 ± 0.18	5.61 ± 0.40
	River-C	7.7 ± 0.03	28.5 ± 0.33	215.83 ± 3.17	1.38 ± 0.13	2.98 ± 0.14
	F	0.790	14.05	111.2	13.94	2.455
	P	0.472	0.0004	0.0001	0.0004	0.119

Keys: *p* = significance level at *p* ≤ 0.05; *p* > 0.05-no significant difference values, *p* < 0.05-significantly different values, *p* < 0.01-highly significantly different values.

The pH values determined at River-A varied between 7.0 and 7.3 during the raining season (July to September), while they varied between 7.2 and 7.8 during the dry season (October to December). Similarly, the pH at River-B varied between 7.0 and 7.5 during the raining season and 7.1 and 8.3 for the dry season, respectively. At River-C, the values obtained for pH ranged between 7.1–74 and 7.5–7.9 for raining and dry seasons. The evaluated pH values were statistically significant during the two seasons (Table 4) at *p* < 0.05, and the values generally fell within the stipulated limits for drinking water according to the SON and the WHO standards (Table 3).

The temperature regimen obtained at all sampling points and across both seasons ranged between 25 and 32 °C. Temperature for most of the sampling period was within the WHO recommended limits of 25–30 °C except at River-A and River-C, where temperatures were slightly above the limits during the dry months of November and December (Table 3). However, the temperature values obtained were, statistically significant at *p* < 0.05 or *p* < 0.01. Likewise, total dissolved solids concentrations at all sampling points complied with the recommended limits of both the SON and WHO (Table 3). The TDS values were statistically insignificant during the two seasons (Table 4).

The determination of the dissolved oxygen in the river water samples showed ranges of 0.4–6.5 mg/L, 1.3–9.3 mg/L, and 0.9–6.2 mg/L at River-A, River-B, and River-C, respectively. However, over the sampling period, only 22.2% (24/108) of the water samples had DO concentrations higher or equal to 5 mg/L for drinking water, as recommended by the WHO (Table 3). The statistical significance test for the DO showed that the values at the three sample sites do not vary significantly at *p* < 0.05. The BOD_5_ values at the three sampling sites ranged between 0.53 and 8.80 mg/L throughout the sampling period. Similar to the DO values, the BOD_5_ values do not vary significantly at* p* < 0.05.

The seasonal statistical analysis of the pH values showed no significant variations across the three study sites. Equally, other parameters, including temperature, DO, and BOD_5_, do not vary significantly (*p* < 0.05) seasonally at the three study sites except TDS, whose values varied significantly during the two seasons.

### Bacteriological characteristics and abundance of the target microbial groups

As presented in Table 5, the prevalence and distribution of the target bacterial groups varied widely in the samples analysed. The one hundred and eight (108) river samples analysed had at least one of the target bacteria. Table 5 shows the presumptive counts of the target bacteria generally ranging from 7.1 × 10^2^ to 1.7 × 10^7^ cfu/100 mL for heterotrophic plate counts; 1.4 × 10^2^ to 8.3 × 10^4^ cfu/100 mL for total coliforms; 1.0 × 10^2^ to 3.1 × 10^4^ cfu/100 mL for faecal coliforms; 1.1 × 10^2^ to 1.4 × 10^4^ cfu/100 mL for *A. hydrophila* and 1.0 × 10^2^ to 4.2 × 10^5^ cfu/100 mL for *E. coli* O157:H7.

**Table 5:** Mean counts of different microbiological parameters on samples collected from the three rivers.

Study site	Bacterial target	Bacteria counts (cfu/100 ml)											
		July ’18		August ’18		September ’18		October ’18		November ’18		December ’18	
		1st	2nd	1st	2nd	1st	2nd	1st	2nd	1st	2nd	1st	2nd
RIVER-A	HPC	1.6 × 10^6^	7.1 × 10^2^	2.3 × 10^4^	4.6 × 10^4^	2.9 × 10^4^	4.4 × 10^5^	4.3 × 10^4^	3.5 × 10^4^	4.8 × 10^5^	7.1 × 10^5^	TNTC	TNTC
	TC	6.3 × 10^2^	6.3 × 10^2^	7.0 × 10^2^	7.0 × 10^2^	1.4 × 10^3^	4.3 × 10^3^	7.6 × 10^3^	9.1 × 10^3^	1.7 × 10^4^	2.6 × 10^4^	4.8 × 10^4^	7.8 × 10^4^
	FC	1.0 × 10^2^	1.2 × 10^2^	2.3 × 10^2^	6.1 × 10^2^	1.4 × 10^3^	1.7 × 10^3^	3.4 × 10^3^	9.5 × 10^3^	8.3 × 10^3^	2.6 × 10^4^	1.0 × 10^4^	1.5 × 10^4^
	*A. hydrophila*	1.2 × 10^2^	1.3 × 10^2^	1.8 × 10^2^	2.1 × 10^3^	1.6 × 10^3^	5.2 × 10^3^	2.7 × 10^3^	6.2 × 10^3^	7.6 × 10^3^	7.8 × 10^3^	1.1 × 10^4^	9.8 × 10^3^
	*E. coli* O157:H7	2.7 × 10^3^	3.0 × 10^3^	1.2 × 10^4^	4.2 × 10^3^	1.2 × 10^4^	7.8 × 10^3^	1.8 × 10^3^	6.3 × 10^3^	1.5 × 10^4^	9.8 × 10^3^	6.0 × 10^3^	1.3 × 10^4^
RIVER-B	HPC	2.9 × 10^3^	9.3 × 10^2^	1.3 × 10^4^	2.2 × 10^4^	2.7 × 10^4^	2.3 × 10^4^	3.3 × 10^4^	7.4 × 10^4^	1.5 × 10^5^	3.2 × 10^5^	TNTC	TNTC
	TC	1.4 × 10^2^	7.4 × 10^2^	6.2 × 10^3^	8.9 × 10^3^	1.3 × 10^3^	1.8 × 10^3^	2.3 × 10^3^	4.3 × 10^3^	9.5 × 10^3^	5.9 × 10^3^	3.6 × 10^4^	4.3 × 10^4^
	FC	1.0 × 10^2^	2.1 × 10^2^	3.6 × 10^2^	3.8 × 10^2^	9.1 × 10^2^	1.9 × 10^3^	1.4 × 10^3^	3.4 × 10^3^	6.2 × 10^3^	3.1 × 10^4^	2.0 × 10^4^	1.4 × 10^4^
	*A. hydrophila*	3.3 × 10^2^	NG	3.5 × 10^2^	4.6 × 10^2^	6.3 × 10^2^	7.1 × 10^3^	7.2 × 10^2^	8.3 × 10^2^	1.0 × 10^3^	1.4 × 10^4^	1.6 × 10^3^	2.5 × 10^3^
	*E. coli* O157:H7	3.3 × 10^2^	1.0 × 10^2^	4.5 × 10^2^	7.2 × 10^2^	7.9 × 10^2^	8.9 × 10^2^	9.8 × 10^2^	8.3 × 10^2^	1.0 × 10^3^	1.4 × 10^4^	2.1 × 10^3^	2.6 × 10^3^
RIVER-C	HPC	7.6 × 10^4^	6.3 × 10^3^	1.9 × 10^4^	5.1 × 10^4^	4.2 × 10^5^	TNTC	TNTC	TNTC	7.5 × 10^6^	8.7 × 10^6^	1.7 × 10^7^	1.7 × 10^7^
	TC	6.5 × 10^2^	3.1 × 10^3^	4.9 × 10^3^	5.7 × 10^3^	1.2 × 10^4^	3.0 × 10^4^	3.5 × 10^4^	5.4 × 10^4^	5.5 × 10^4^	8.3 × 10^4^	7.2 × 10^4^	8.3 × 10^4^
	FC	3.0 × 10^1^	1.4 × 10^2^	2.2 × 10^2^	8.1 × 10^2^	1.1 × 10^3^	1.9 × 10^3^	2.7 × 10^3^	3.9 × 10^3^	5.8 × 10^3^	6.2 × 10^3^	1.3 × 10^4^	7.8 × 10^3^
	*A. hydrophila*	3.4 × 10^3^	2.3 × 10^2^	1.1 × 10^2^	4.2 × 10^2^	5.6 × 10^2^	7.8 × 10^2^	2.1 × 10^2^	9.8 × 10^2^	1.5 × 10^3^	3.2 × 10^3^	4.0 × 10^3^	7.4 × 10^3^
	*E. coli* O157:H7	3.4 × 10^3^	2.3 × 10^3^	1.4 × 10^3^	3.3 × 10^3^	7.1 × 10^3^	7.8 × 10^3^	1.4 × 10^3^	7.9 × 10^3^	4.2 × 10^5^	9.8 × 10^3^	9.5 × 10^3^	1.3 × 10^4^

*HPC* heterotrophic plate count, *TC* total coliforms, *FC* faecal coliforms, *TNTC* too numerous to count, *NG* no growth.

The lowest HPC count was observed during the raining season in July 2018 at River-A, while HPC density was too numerous to count (TNTC) during the dry season at the same sites. For the coliforms, the lowest count of TC (1.4 × 10^2^ cfu/100 mL) was observed during the raining months of July at River-B, while the lowest count of FC was observed at River-C during the raining season. The highest concentrations of TC (8.3 × 10^4^ cfu/100 mL) and FC (3.1 × 10^4^ cfu/100 mL) occurred during the dry season at River-C and River-B, respectively (Table 5). *A. hydrophila* had the least count of 1.1 × 10^2^ cfu/100 mL at River-C in August 2018 while the highest density of *A. hydrophila* (1.4 × 10^4^ cfu/100 mL) was recorded at River-B in November 2018. Presumptive *E. coli* O157:H7 was detected in all the samples analysed, with the lowest count (1.0 × 10^2^ cfu/100 mL) occurring at River-B in July 2018 and the highest count in November, also at the same river.

Statistical comparison of the mean values from the three rivers across both seasons showed that the counts exceeded the standard limit of 0 cfu/100 5 mL of coliforms for portable and water as recommended by the WHO, the SON, and the FMEnv^34–36^. Also, the mean values of the microbial populations for the samples collected at all three sites during the dry months generally exceeded those collected during the raining months, with highly significant differences for microbiological parameters such as HPC (*p* = 0.0013), TC (*p* = 0.0019), FC (*p* = 0.0008) and *A. hydrophila* (*p* = 0.0012). Similarly, there is a slightly positive correlation between the physicochemical characteristics of the rivers and the microbiological qualities. For instance, increased temperatures, mostly observed during the dry months, appear to favour the microbial population densities of HPC, TC, FC, and *A. hydrophila*. Also, pH exhibited a strong positive correlation (r = 0.9219) with FC densities. However, an inverse correlation generally existed between the microbial density and dissolved oxygen in the samples.

### Biochemical characterisation of presumptive isolates

Seventy-six (76, 18.1%) isolates, out of 420 presumptive isolates from the analysed water samples, exhibited typical aeromonad biochemical characteristics. Of these, 44.1% (34/76) was recorded from River-A, while River-B and River-C had 30.6% (23/76) and 25.3% (19/76), respectively. Similarly, 15.5% (65/420) of the presumptive isolates showed typical *E. coli* biochemical characteristics with recovery rates as follows: River-A (26.2%; 17/65), River-B (27.7%; 18/65) and River-C (46.2%; 30/65).

### Antimicrobial resistance profile of A. hydrophila and E. coli O157:H7 isolates

The antibiotic resistance profile of the one hundred and forty-one (141; 76 *A. hydrophila* and 65 *E. coli* O157:H7) identified isolates and the multiple antibiotic resistance indices at the different sampling sites are presented in Table 6. The tested isolates exhibited more than 80% (≥ 80%) resistance against five of the ten test antimicrobials, with resistance against cefixime, a cephalosporin antibiotic being the highest at 95% (134/141), followed by 94.3% (133/141) resistance against cefuroxime (a second-generation cephalosporin), 92.9% (131/141) resistance against augmentin, 90.8% (128/141) against cefotaxime and 87.2% (123/141) resistance against amoxicillin. Generally, *A. hydrophila* isolates showed higher frequencies of resistance against the test antimicrobials, with all 76 (100%) completely resistant against cefuroxime and cefotaxime and with MARI ≥ 0.61 (Table 6). Similarly, isolates from River-A generally tend to show more resistance against the test antimicrobials than isolates from the other two sampling sites, with MARI = 0.68 for *A. hydrophila* and MARI value of 0.059 for *E. coli* O157:H7 (Table 6). All the test isolates from this study exhibited multiple antibiotic resistance phenotypes (MARP), with resistance against three or more of the test antimicrobials. The commonest MARP among the *A. hydrophila* isolates is CAZ-CXM-CIP-GEN-NIT-OFL, occurring in 14 (18%) isolates while, 5 isolates were completely resistant against the 10 test antimicrobials. Three (3) of the *E. coli* O157:H7 isolates also exhibited complete resistance against all test antimicrobials, while MARP 7 was the most frequently encountered multiple resistant patterns among the *E. coli* isolates with about 33.3% showing resistance against 7 of the test antimicrobials.

**Table 6:** Percentage resistance *A. hydrophila* and *E. coli* O157:H7 to selected antibiotic and MAR index for selected rivers.

Sampling site	Test bacteria	Number of test isolates	Number of resistant isolates (%)										
			Test antibiotics										
			AUG	CTX	CXM	CAZ	CFM	OFL	NIT	GEN	AMX	CIP	MARI
River-A	*A. hydrophila*	24	24 (100)	24 (100)	24 (100)	14 (58.3)	23 (95.8)	2 (8.3)	13 (54.2)	8 (33.3)	23 (95.8)	0	0.68
	*E. coli* O157:H7	17	15 (88.2)	15 (88.2)	12 (70.6)	10 (58.8)	17 (100)	17 (100)	15 (88.2)	17 (100)	13 (76.5)	2 (11.8)	0.059
River-B	*A. hydrophila*	18	18 (100)	18 (100)	18 (100)	11 (61.1)	17 (94.4)	1 (5.6)	10 (55.6)	5 (27.8)	18 (100)	0	0.61
	*E. coli* O157:H7	18	14 (77.8)	13 (72.2)	16 (88.9)	14 (77.8)	17 (94.4)	15 (83.3)	14 (77.8)	8 (44.4)	12 (66.7)	7 (38.9)	0.055
River-C	*A. hydrophila*	34	33 (97.1)	34 (100)	34 (100)	27 (79.4)	30 (88.2)	6 (17.7)	25 (73.5)	16 (47.1)	31 (91.2)	0	0.61
	*E. coli* O157:H7	30	27 (90)	24 (80)	29 (96.7)	28 (93.3)	30 (100)	28 (93.3)	28 (93.3)	24 (80)	26 (86.7)	19 (63.3)	0.033
Total		141	131 (92.9)	128 (90.8)	133 (94.3)	104 (73.8)	134 (95)	69 (48.9)	105 (74.5)	78 (55.3)	123 (87.2)	28 (19.9)	

*AMX* amoxicillin, *AUG* augmentin, *CFM* cefixime, *CTX* cefotaxime, *CAZ* ceftazidime, *CXM* cefuroxime, *CIP* ciprofloxacin, *GEN* gentamicin, *NIT* nitrofurantoin, *OFL* ofloxacin, *MAR* multiple antibiotic resistance index.

Likewise, 89.5% (68/76) of the *A. hydrophila* isolates showed phenotypic ESBL production from the double-disc diffusion assays, while 38 (58.5%) of the *E. coli* isolate showed ESBL production phenotypically.

### Molecular characterization of antibiotic resistance and virulence genes in isolates

The genotypic characterization of the isolates by PCR assays revealed the presence of the three ESBL-producing genes selected for this study, as shown in Table 7. The frequency of the detection of the resistance genes in the *A. hydrophila* isolates generally ranged between 0% (*bla*_SHV_) and 26.3% (*bla*_CTX-M_), while the frequency of detection among the *E. coli* O157:H7 isolates ranged between 4.6% (*bla*_CTX-M_) and 58.4% (*bla*_TEM_). Some of the representative gel electrophoretic images of the detected genes are shown in Supplemental Fig. 1a–c. Among the 65 *E. coli* O157:H7 isolates, 32.3% (21/65) harboured the flic_H7_ gene, while 21.5% (14/65) harboured the rfbE _O157_ gene. However, the hemolysis-producing (*hyl*A) gene was detected in 12% (9/76) of the isolates. The presentative gel electrophoretic images of the amplified virulence genes are presented in Supplemental Fig. 2a–c.

**Table 7:** Detection of ESBL-producing genes in *A. hydrophila* and *E. coli* O157:H7 isolates.

ESBL gene	Amplicon size	Test bacteria	Number of isolates tested	Number of positive isolates
*bla*_CTX-M_	585 bp	*A. hydrophila*	76	20 (26.3%)
		*E. coli* O157:H7	65	3 (4.6%)
*bla*_SHV_	393 bp	*A. hydrophila*	76	2 (2.6%)
		*E. coli* O157:H7	65	12 (18.5%)
*bla*_TEM_	517 bp	*A. hydrophila*	76	0
		*E. coli* O157:H7	65	38 (58.5%)

## Discussion

Rivers are waterways of strategic importance for the sustenance of life and economic growth across the globe. Indiscriminate pollution and declining freshwater quality threaten human health and economic development and is a cause for concern. Proper maintenance of a healthy aquatic ecosystem depends on the physicochemical characteristics and biological diversity, which have interdependent effects on each other^37^. Regular and proper monitoring of waterbodies will not only assist in preventing disease outbreaks but also forestall further water quality deterioration towards human health and environmental protection.

Even though the water samples analysed in this study complied with the recommended standards in terms of the physicochemical characteristics and for most of the sampling period, there were certain instances where the water quality did not comply with the set limits. For instance, as depicted in Table 4, only approximately 22.2% of the water samples analysed had dissolved oxygen within the recommended limit set by the WHO (Table 3). Dissolved oxygen is a vital component that determines the quality of a waterbody and the trophodynamics of an aquatic ecosystem. The concentration of dissolved oxygen close to saturation implies a relatively healthy water system, while the United States Environmental Protection Agency (U.S. EPA) recommends an oxygen concentration exceeding the chronic criterion for growth (4.8 mg/L) for a healthy water ecosystem^38^. Low dissolved oxygen levels below the criterion may lead to a deleterious effect on the health of the aquatic ecosystem, such as anoxia or hypoxia conditions, which in turn may result in the alteration of the ecosystem balance. Apart from anthropogenic influences, other factors, such as atmosphere-water surface exchange, nitrification, sediment–water exchange, mineralisation, respiration, and photosynthesis, affect the dissolved oxygen dynamics of an aquatic system^39^. The relatively low dissolved oxygen observed in some samples may be due to the oxygen-demanding substances. The dissolved oxygen regime observed in the study is similar to those reported elsewhere^40,41^.

Biological oxygen demand assessment is another useful characteristic in measuring the compliance of water systems with set limits and the estimation of dissolved or biodegradable organics that may deplete oxygen^42^. The BOD_5_ regime of the samples was mostly in line with the recommended limits or ≤ 6 mg/L except on a few occasions (Table 4). The non-compliance of the river water to the limits in some instances may be due to the release of some oxygen-demanding substances into the river, which may be due to numerous small-scale industrial activities around the river courses.

Enteric bacteria are some of the mostly encountered microorganisms in aquatic systems. This study investigated the abundance of enteric bacteria and some important gastroenteritis pathogens in the water samples from three rivers. More than 97% of the water samples analysed in this study had HPC counts higher than the WHO standard of 100 cfu/ml (Table 5), while significantly higher bacterial counts were generally observed during the drier months than the raining months. HPC or standard plate count includes all bacteria, mould, and yeast that require organic carbon source and certain inorganic salts for their growth. Although some researchers have divided opinions on the importance of HPC measurement in water systems, it is generally believed that increases in HPC bacteria may be an early indicator of pollution. The progressive rise of HPC may have implications for public health, particularly among immunocompromised individuals and may signal the likely presence of opportunistic pathogens, which may cause gastroenteritis, diarrhoea and other related intestinal infections^43^. Our results are comparable to those reported elsewhere^44^.

Total coliforms (TC) are a broad range of collectively harmless bacteria found mainly in the gastrointestinal tract of humans, other warm-blooded animals, and the environment. A particular subset of the group is the faecal coliforms (FC), with the most frequent member being *Escherichia coli*. The FCs are mainly separated from other coliforms by their ability to grow at elevated temperatures (44.5 °C) with or without oxygen. Similar to the trend observed for HPC, coliform bacteria counts were generally higher during the dry months of October to December, as seen in Table 5. The highest count of TC (8.3 × 10^4^ cfu/100 mL) was observed at River-C in December, while the lowest count occurred at River-B in July. The highest density of FC (3.1 × 10^4^ cfu/100 mL) was recorded in November at River-B, while the least count was in July '18 at River-C (Table 5). Although coliforms themselves are generally not pathogenic, their presence indicates faecal contamination of water by human or other warm-blooded animals’ faecal matter. Their presence also indicates the possible presence of pathogens and other parasites, which may be associated with diseases such as typhoid fever, viral and bacterial gastroenteritis, and hepatitis A, among others^45^. Faecal contamination of water presents a potential risk for human exposure. As observed in our results, coliform bacteria might have resulted from the overflow of domestic sewage and other nonpoint sources of anthropogenic and agricultural waste materials. Counts observed in this study mostly complied with the WHO limit of 10^3^ cfu/100 mL during the raining months, which may be a result of the dilution effect of rain around this time of the year, while counts were generally higher than the limits during the drier months. Our findings are similar to those reported elsewhere^44,46^.

Motile *Aeromonas* species are opportunistic pathogens isolated frequently from polluted water sources with different degrees of contamination. However, various reports have identified *Aeromonas* as an aetiological agent of human enteric infections^47–49^. Typical faecal indicator bacteria monitoring may not adequately assess the public health risk associated with opportunistic pathogens such as *Aeromonas*. In the current study, *Aeromonas* spp. populations at the 3 study sites ranged between 1.2 × 10^2^–9.8 × 10^3^ cfu/100 mL at River-A, 3.3 × 10^2^–1.4 × 10^4^ cfu/100 mL at River-B and 1.1 × 10^2^–7.4 × 10^3^ cfu/100 mL at River-C. Wide variations were generally observed in the distribution of *Aeromonas* spp. in the water samples, with densities mostly increasing in the drier months as compared to the raining months (Table 5). Reduced rainfalls and more stable temperatures may contribute factors to the general increase in bacterial densities during the dry months^50,51^. Seventy-six (18.1%) of the 420 randomly picked isolates were identified as *Aeromonas* spp. by physiological and conventional biochemical analyses. According to the test, the 76 isolates were Gram-negative, motile, rod-shaped, glucose-fermenting, and facultative anaerobes. The isolates were also oxidase, catalase, and indole positive while negative for the urease test (Table 6). *Aeromonas* spp. have become important emerging human pathogens causing wound infection, septicaemia and diarrhoeal illnesses^52,53^. The prevalence and distribution of *Aeromonas* species in aquatic systems, their roles as microbial pollutants for surface and drinking water supplies, and their ability to induce diseases are of great concern to public health.

Cattle are the main reservoirs of *E. coli* O157:H7, which they typically excrete at 10^2^ to 10^5^ colony-forming cfu per gram of faeces^54,55^. *E. coli* O157:H7 bacteria commonly exist in the intestinal tracts of cattle and may find their way into waterways through agricultural organic waste runoffs or direct deposition of faecal matter into surface waters. Surface water close to farms may thus represent a potential reservoir for enteric pathogens, including *E. coli* O157:H7, which may allow re-infection cycles and increasing the potential for the pathogens to spread^56^. *E. coli* O157:H7 was detected in all the samples collected from the three study sites selected for this study, with presumptive counts ranging from 2.7 × 10^3^ to 1.5 × 10^4^ cfu/100 mL, 1.0 × 10^2^ to 1.4 × 10^4^ cfu/100 mL and 2.3 × 10^3^ to 4.2 × 10^5^ cfu/100 mL at River-A, River-B, and River-C respectively. The detection of *E. coli* O157:H7 bacteria from all the study sites suggests that the rivers may serve as reservoirs for this pathogen and may be a significant source of dissemination of *E. coli* O147: H7 in the environment and subsequently to humans via direct contact with contaminated water sources and indirectly via the food chain. *E. coli* O157:H7 have been reported to survive for about two months in rivers, lakes, and animal trough water samples^56^. Enterohaemorrhagic *E. coli* strains (*E. coli* O157:H7) has a low infectious dose of about 20 to 700 cell and can cause life-threatening haemorrhagic colitis and haemolytic uraemic syndrome. Some strains can produce highly potent cytotoxins similar to the Shiga toxin produced by *Shigella* species, which causes dysentery^57^. The United States Centers for Disease Control and Prevention (CDC) estimated the annual economic burden of *E. coli* O157:H7-related illnesses due to hospitalization and other medical expenses as well as the loss of life and productivity at about $405 million, while the annual illnesses associated with *E. coli* O157:H7 in the United States alone is estimated at 63,000 with over 2100 hospitalizations and 20 deaths^58,59^. Although there is currently no maximum contaminant level (MCL) for specific *E. coli* strains such as *E. coli* O157:H7, the United States Environmental Protection Agency (EPA) included *E. coli* O157:H7 on the Contaminant Candidate List 3 (CCL3) of September 21, 2009. The CCL3 includes contaminants currently unregulated, known, or anticipated to occur in public water supplies and may require regulation under the Safe Drinking Water Act^60^. The *E. coli* O157:H7 counts obtained at the various sites in this study did not correlate with coliform counts and generally fell short of the coliforms recommended limits, which suggests the unsuitability of the water sources for direct domestic use or other contact activities. As recorded in this study, high levels of microbiological pollution indicators were generally observed in samples from the three study sites, mostly during the dry seasons might have resulted from the intense anthropogenic activities around the study sites, as described in Table 1.

In the current study, the identified *Aeromonas* spp. and *E. coli* O157:H7 isolates subjected to antibiotic susceptibility testing (AST) showed increasing resistance towards 3 antibiotics (augmentin, 97.1%, cefotaxime, 100%, and cefuroxime, 100%) out of a panel of 10 conventional antibiotics used for the test (Table 6). Overall, the highest incidences of antibiotic resistance were observed against cefixime, with a frequency of 95% (134/141), while the least resistance was against ciprofloxacin, at a frequency of 19.9% (28/141). Antimicrobial resistance poses a serious global public health threat because of the continuous emergence and spread of multidrug-resistant “superbugs”. The annual estimate of illnesses associated with antimicrobial-resistant pathogens is approximately 700,000 globally, while deaths associated with antimicrobial resistance have been projected to reach 10 million, with a global loss of about US$100 trillion by the year 2050^61,62^. Indiscriminate use of antimicrobials remains a significant factor contributing to the proliferation of antimicrobial resistance. Our finding contradicts the report of^63^, who reported 100% resistance against ciprofloxacin while researching the resistance profiles of *Aeromonas* isolates from water-related wound infections. Contrarily, our observation is corroborated by the report of Koksal et al.^64^, who reported only 1% resistance to ciprofloxacin for *Aeromonas* strains isolated from drinking water samples in Istanbul, Turkey.

The multiple antibiotic index (MARI) threshold of 0.2 is used to determine the possible exposure of isolates to antibiotic resistance selective pressures such as antimicrobial and heavy metals in their environment^26^. The observed MARIs in this study generally show *Aeromonas* spp. having MARI values (Table 6) above the threshold at all the sampling points, while *E. coli* O157 isolates were mostly below the threshold. This might have resulted from the occurrence of numerous *Aeromonas* isolates with high multidrug resistance phenotypes, as observed in the AST analysis in this study.

Extended-spectrum beta-lactamases (ESBLs) are important enzymes produced by bacteria which hydrolyze and inactivate beta-lactam antibiotics. Since their initial discovery in the 1980s, they have increased in prevalence among bacterial pathogens. The production of ESBL is a significant health concern because they reduce or destroy the potency of common drugs, including penicillins, carbapenems and cephalosporins, thus making infections more difficult to treat with increased complications^65^. In this study, a high prevalence (89.5%; 68/78) of phenotypic ESBL production was observed among the *Aeromonas* isolates from the water samples, and *E. coli* O157:H7 has a prevalence of 58.5% (38/65). ESBL-producing bacteria have increasingly been reported in clinical and environmental isolates in recent years, with three major classes of chromosomally-mediated β-lactamase–Amber class B, C and D being recognized^66,67^. The actual prevalence of ESBL-producing bacteria is believed to be underestimated because of the various challenges associated with their identification in clinical laboratories^68^. The prevalence of ESBLs in Africa has mostly been reported from human and animal samples (between 11–72%), while current reports from environmental samples (7–79%) are mostly from wastewater^69^. This current study reports ESBL prevalence in *Aeromonas* species and *E. coli* O157:H isolates recovered from freshwater sources which may present a health risk to the communities and persons in direct contact with water from the sources. Our observation is significant because of the scarcity of documented information on the prevalence of ESBL-producing *Aeromonas* species and *E. coli* O157:H7 in rivers in the southwest region of Nigeria. Our observation is similar to the study of Banu et al.^70^, who reported a prevalence of 98% (94/96) for ESBL-producing *E. coli* from river water samples in Ghana.

The molecular characterisation of the ESBL genotypes showed that most of the ESBL-producing *Aeromonas* spp. harboured the *bla*_CTX-M_ gene**,** which was detected in 26.3% (20/76) isolates analysed by PCR, while the *bla*_TEM_ gene was not detected in any of the *Aeromonas* isolates and 2.6% (2/76) harbouring the *bla*_SHV_ gene (Table 7). In contrast, most of the ESBL-producing *E. coli* O157:H7 isolates harboured the *bla*_TEM_ gene 58.5% (38/65), while the *bla*_SHV_ gene was detected in 18.5% (12/65) of the isolates and *bla*_CTX-M_ detected in 4.6% (3/65) isolates. The majority of molecular studies worldwide have reported the *bla*_TEM_ gene as the most commonly encountered ESBL genotype^71–73^. This coincides with our observation among the *E. coli* O157:H7 analysed in this study; however, the *bla*_TEM_ gene was not detected among the *Aeromonas* isolates from this study.

The *bla*_TEM_ genes are among the most clinically relevant beta-lactamases due to their ability to produce enzymes with broad-spectrum activities that hydrolyse the cyclic amide bond of the beta-lactam rings, including those of penicillins and cephalosporins. The genes were initially thought to be chromosomal; however, they have been identified on plasmids, and their mobility has been associated with transposon and integrons^74^. Similarly, the *bla*_SHV_ genes have become clinically important because of their ability to hydrolyse a broad spectrum of antibiotics by causing changes to the configurations of the active sites of drugs, including monobactam and carbapenems. The group currently encompasses a large number of allelic variants which can be exchange between pathogens via horizontal gene transfer^75^. Mobile genetic elements (including plasmids and integrons) and epidemic strains of *Enterobacteriaceae* have been mostly associated with the widespread and success of the *bla*_CTX-M_ genes. The mobile genetic elements are been identified to provide the conditions for the persistence of this gene among bacteria through the co-location of multiple resistance determinants and the co-selection by several antimicrobials^76^.

The PCR amplification of virulence genes in the isolates revealed the presence of *hyl*A, one of the genes involved in haemolysin production in 9 of the *E. coli* O157:H7 isolates. Enterohaemorrhagic *E. coli* (EHEC) is known to harbour other putative virulence genes, such as *stx1* and *stx2*. Two genes (rfbE_O157_ and fliC_H7_) were also detected in 21 and 14 *E. coli* O157:H7 isolates. The gene rfbE_O157_ encodes the *E. coli* O157:H7 somatic antigen O157, while the fliC_H7_ encodes the structural flagella antigen designated H7. Both of these genes provide molecular identification of the O157:H7 *E. coli* serotype and can be used to differentiate the highly virulent serotype from *E. coli* strains^33^.

In conclusion, direct or indirect contact with polluted water sources can lead to an outbreak of infectious diseases contributing to the increased burden of enteric and extra-intestinal diseases. Attempts at providing safe, clean, and adequate water supplies will not safeguard human health and promote socioeconomic development and human dignity. Although the findings of this study showed that some physicochemical characteristics of the sampled rivers were within the acceptable limits, even so, those characteristics that do not comply with the set limits cannot be ignored. In the same vein, the bacteriological quality of the samples suggests potential human health and environmental risk. The detection of potentially pathogenic and multidrug-resistant *Aeromonas* and *E. coli* strains and high levels of indicator bacteria reflects the possible threat associated with direct contact or ingestion of water from these freshwater sources without adequate treatment. One major significant implication regarding the detection of the virulence and antimicrobial resistance genes in isolates from these freshwater sources is their possible persistence and spread to other potential pathogens not targeted in this study. Therefore, this necessitates effective intervention for managing and improving these important freshwater resources in the interest of public health and environmental protection.

## Supplementary Information

Supplementary Information.

## Data Availability

The authors confirm that the data supporting the findings of this study are available within the article.
