# Supplementary material for: Isolation and genotypic characterization of extended-spectrum beta-lactamase-producing Escherichia coli O157:H7 and Aeromonas hydrophila from selected freshwater sources in Southwest Nigeria
Source: Sci Rep. 2023 Jul 3;13:10746. doi: 10.1038/s41598-023-38014-y (PMC10318038; doi:10.1038/s41598-023-38014-y)

Supplementary information

**Supplemental Figure 1a:** Lane M: 100 bp Molecular ladder; Lane N: Negative control; Lane 1 to 10 isolates positive for *bla*_CTX-M_ (585 bp).

**M N 1 2 3 4 5 6 7 8 9 10**

**300 bp**

**585 bp**

**600 bp**

**500 bp**


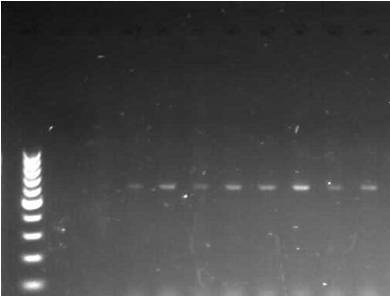

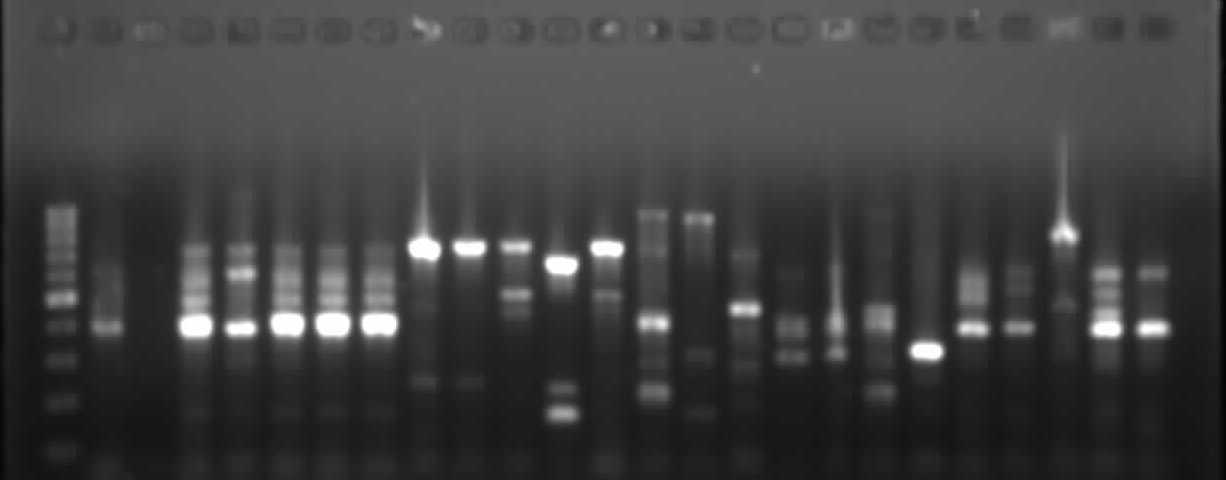


**M 1 N 2 3 4 5 6**

**300 bp**

**500 bp**

**393 bp**

**393 bp**

**Supplemental Figure 1b:** Lane M: 100 bp Molecular ladder; Lane 1: Positive isolate; Lane N: Negative control; Lane 2 to 6 isolates positive for *bla*_SHV_ (393 bp).


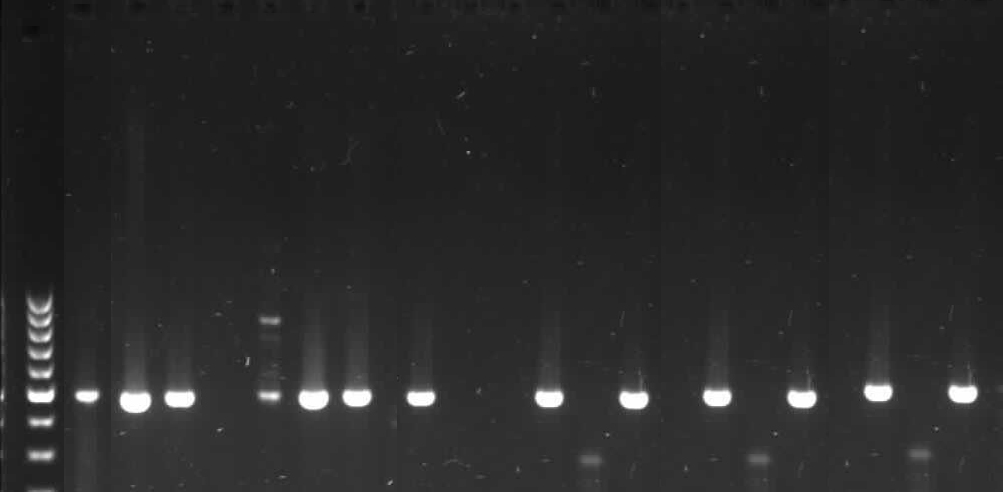


**517 bp**

**500 bp**

**300 bp**

**M 1 2 3 4 5 6 7 8 9 N**

**Supplemental Figure 1c:** Lane M: 100 bp Molecular ladder; Lane 1 to 9 isolate (*bla*_TEM_ 393 bp); Lane N: Negative control.

**
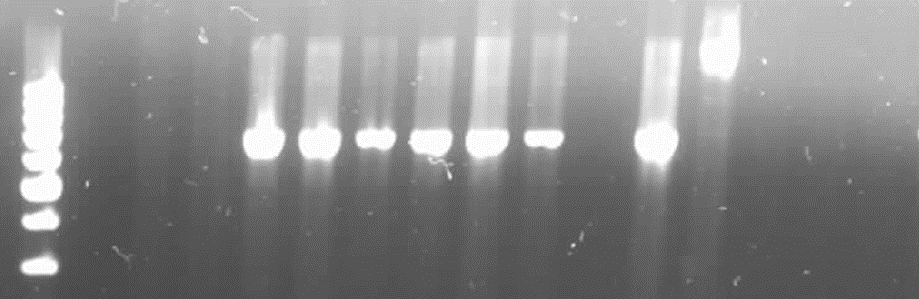
**

**Supplemental Figure 2a:** Lane M: 100 bp Molecular ladder; Lane 1 to 9 isolate (flic_H7_ 625 bp); Lane N: Negative control.

**M N 1 2 3 4 5 6 7 8 9 10**

**600 bp**

**500 bp**

**625 bp**

**Supplemental Figure 2b:** Lane M: 100 bp Molecular ladder; Lane 1 to 9 isolate (rfbE_O157_ 327 bp); Lane N: Negative control.

**M 1 2 3 4 5 6 7**

**327 bp**

**500 bp**

**300 bp**


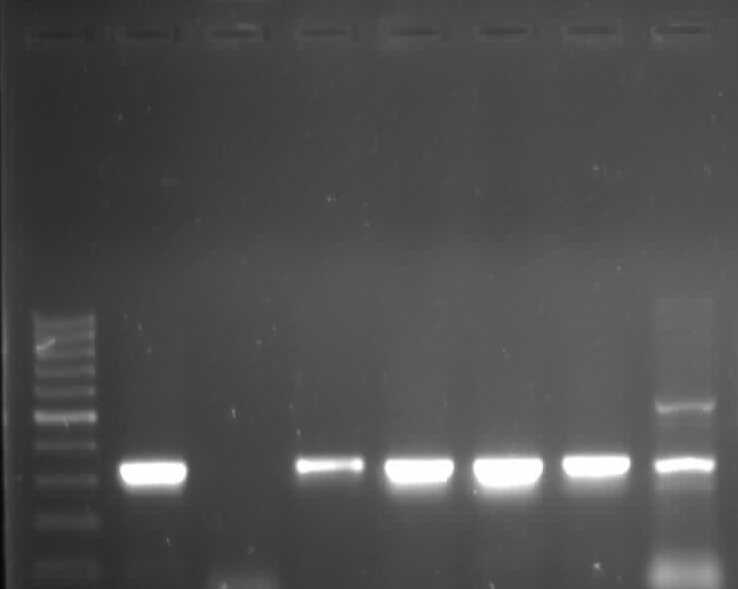


**Supplemental Figure 2c:** Lane M: 100 bp Molecular ladder; Lane 1 to 9 isolate (*hly* 496 bp); Lane N: Negative control.

**M N 1 2 3 4 5 6 7 8 9 10**

**496 bp**

**500 bp**

**300 bp**


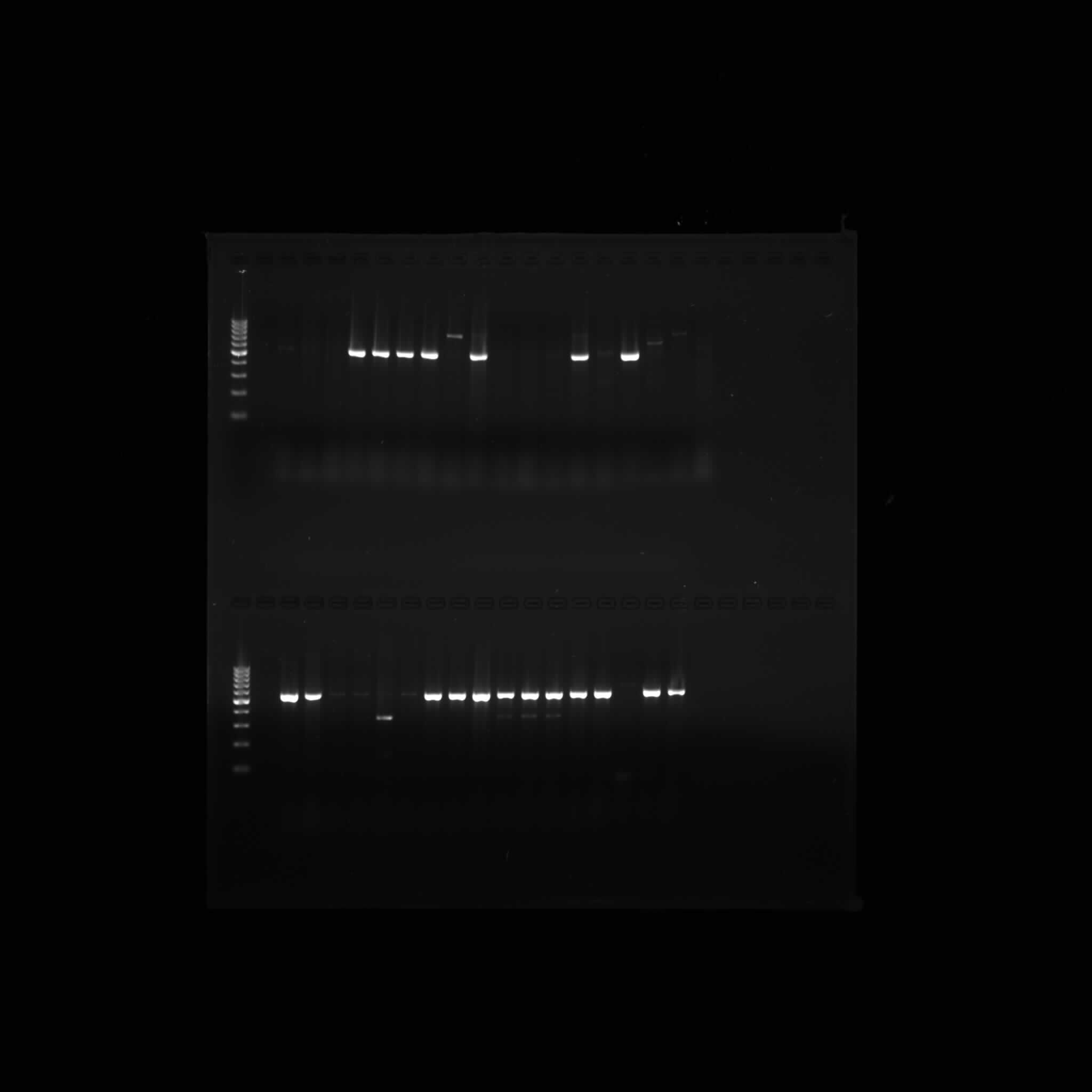

Supplement: Supplementary file 1 — Supplementary Information. [file 41598_2023_38014_MOESM1_ESM.docx]
